# Effect of over the counter mouthwashes with and without alcohol on sorption and solubility of bulk fill resins

**DOI:** 10.4317/jced.57234

**Published:** 2020-12-01

**Authors:** Vera Prado, Karen Santos, Raíssa Fontenele, Joseane Soares, Glauber Vale

**Affiliations:** 1 DDS, MsC, PhD. Restorative Dentistry Department, Federal University of Piauí, Teresina, Piauí, Brazil; 2 DDS. Restorative Dentistry Department, Federal University of Piauí, Teresina, Piauí, Brazil

## Abstract

**Background:**

Composites sorption and solubility can be precursors of several chemical and physical processes, which lead to deleterious effects on the polymer structure. This study evaluated the effect of mouthwashes with and without alcohol on the sorption and solubility of conventional and low viscosity bulk fill resins.

**Material and Methods:**

Four types of Bulk Fill resins (Filtek™ Bulk Fill, X-tra Fil, Filtek™ Bulk Fill Flow and X-tra Base) were submitted to the following mouthwashes: Listerine Cool Mint and Periogard (containing alcohol) and Listerine Zero and Periogard (alcohol-free). The specimens were stored in the mouthwashes for seven days. Solubility and sorption tests were performed according to ISO 4049. Data were analyzed using two-way-ANOVA, followed by Tukey Test. The data were grouped, and a paired t-test was performed to evaluate the effect of alcohol on the properties studied. The p was fixed at 5%.

**Results:**

Resins immersed in alcohol-containing mouthwashes had higher values of sorption and solubility, with the highest sorption rate for X-Tra Base in Listerine Cool Mint treatment (*p*<0.05). Flow type resins showed higher sorption than conventional viscosity resins, irrespective of the mouthwash used (*p*<0.05).

**Conclusions:**

Alcohol-containing mouthwashes affected sorption and solubility of bulk fill resins and the composites that presented worse and better performance regarding the studied properties were X-Tra Base and Filtek™ Bulk Fill, respectively.

** Key words:**Solubility, Sorption, Mouthwashes, Bulk-fill composites.

## Introduction

Resin-composites have been commonly used in restorative dentistry over the years. The conservative preparations and restorations, improved adhesiveness and aesthetics expanded their use as for small cavities as for direct restoration of extensively damaged teeth (1). Their properties have been improved to increase the stability in the oral environment, however, they still have a susceptibility to some chemical degradations, including sorption and solubility in water and other liquids (2).

Ideally, composites must be highly sTable and impermeable to water; however, the dimensional stability of these resins, whose matrix is based on Bis-GMA and Bis-EMA, is compromised due to its hydrophilicity (3-5). Their polymer networks absorb different chemicals from the oral environment and may release some of their components (6). Therefore, sorption and solubility can lead to harmful biological and physical effects on the material (7-10).

Apart from saliva, other solvents are constantly reacting in the oral environment and may result in deleterious effects on the composite resin matrix (11), such as mouthwashes, whose formulation consists of water, antimicrobial agents, salts and in some cases, alcohol (12). Indeed, alcohol is considered as being a good solvent for the polymer chain of the composite resin and when it is found in high concentrations, it can reduce mechanical properties and favour the wear of restorative material (12,13).

Bulk Fill resins can be found in two different formulations that are related to their consistency: low viscosity (flow) and conventional viscosity. In order to achieve the increase in polymerisation depth, manufacturers modified the translucency/opacity of the composite and decreased the number of inorganic particles since light penetration is closely related to particles (14). Also, photoinitiators with greater light absorption were added to the composition of the resins to allow adequate conversion of monomers into polymers, even if inserting increments of 4mm.

Such chemical changes can affect the quality of the polymer network of these materials and their resistance to moisture compared to conventional resin composites. Although many studies have been done with bulk fill resin to investigate its polymerisation, curing depth (15) and its physical, chemical and mechanical performance (16), the literature still lacks data on its stability in long-term aqueous media (5,17).

Thus, considering that the resistance of the material to the challenges of the oral environment is essential for the longevity of the restorations, this study aimed to compare the effect of mouthwashes with and without alcohol on the sorption and solubility of bulk fill resins.

## Material and Methods

-Restorative Materials and solutions 

Four types of low shrinkage (bulk fill) composite resins were used: two with conventional viscosity (Filtek™ Bulk Fill, X-tra Fil) and two with low viscosity (Filtek™ Bulk Fill Flow, X-tra Base), whose basic description is in Table 1. The solutions used (Table 2) consisted of four types of mouthwashes routinely used for oral hygiene, two containing alcohol (Listerine Cool Mint, and Periogard) and two without alcohol (Listerine Zero and Periogard, without alcohol).

**Table 1:**
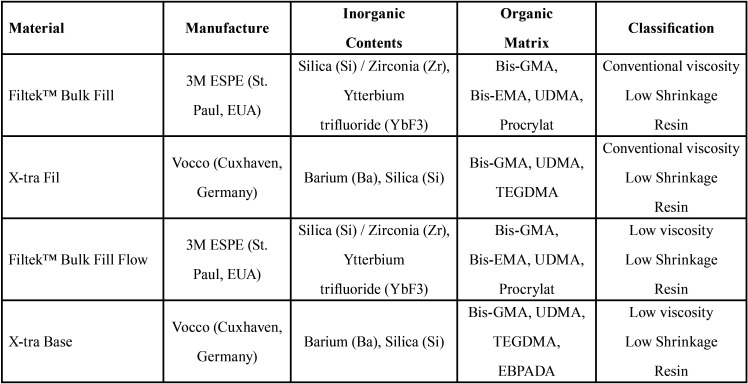
Restorative materials used according classification, manufactures and composition.

**Table 2:**
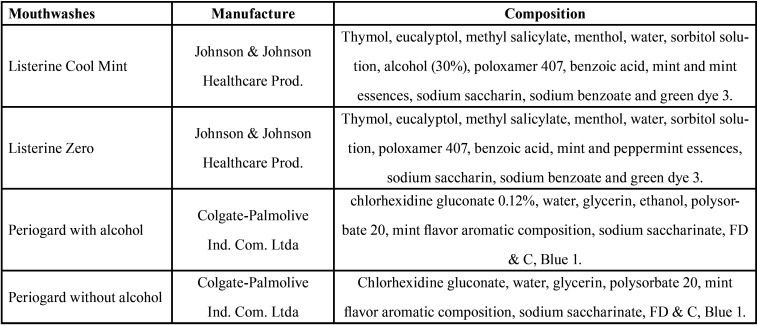
Composition of Mouthwahes used in this study.

-Preparation of Specimens 

**Figure 1:**
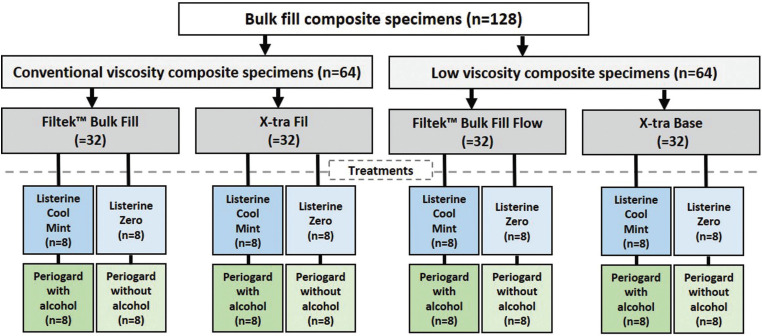
Flow-diagram of composite resin specimens distribution among the types of resin and mouthwashes treatments.

Thirty-two specimens of each composite were randomly divided into four groups (n = 8), totalling 128 units for the whole experiment. The sample size was determined based on previous findings using the same experimental protocol (18), with a statistical power higher than 0.9. The specimens were obtained using a Teflon mold containing 6 circular perforations (4mm diameter x 2 mm thick). The composite resin insertion inside the Teflon matrix was performed in a single increment. A polyester strip was placed and a glass slide (weighing 272g) was pressed for 10 seconds against the material to remove excess and against the surface of each specimen to acquire a smooth and flat appearance. After this, 40 seconds of photoactivation was applied according to the manufacturer’s specifications. After this time, the specimens were removed from the matrix and placed in labelled test tubes. The composites were light-polymerized using a halogen-based light-curing unit (Optilux 400-DemetronResearch Corporation, Danbury, CT, USA). The light output was tested (480 ± 32 mW/cm2) before each use with a Demetron Model 100 radiometer (Demetron Research Corporation, Danbury, CT, USA). Subsequently, the samples were polished with sandpaper discs (Soft flex TDV) under low speed to remove the excesses and the debris was removed with a light jet of air. The distribution of specimens among the types of composites and treatments is shown in Figure 1.

-Sorption and Solubility Measurements 

The measurement of the composite resins sorption and solubility was performed in accordance with ISO 4049 (19). The test specimens were stored in a desiccator with blue silica gel and after 24 hours were weighed on a calibrated analytical balance with high precision (± 0.00001 mg, Shimadzu, Japan) to obtain a stable initial weight. This cycle was repeated 24 hours until a constant mass (m1) was observed. After stabilisation of the initial mass, diameter and thickness of the specimens were measured using a digital calliper (± 0.001 mm) by a single trained examiner. The diameter of each sample was measured at two points perpendicular to one another and the average diameter was calculated. The thickness of each specimen was measured at the centre of the same four equally spaced points and average thickness was calculated. To calculate the volume (V) of the specimen, the following formula was used: V = π x r2 x h, where r is the radius of the average (diameter/2) and h is the average thickness.

After determining the volume of the test specimens, they were stored separately in 2 mL of each solution for seven days, with the solution being changed daily. After this period, the samples were removed with tweezers, abundantly washed with distilled water and dried with an absorbent paper towel, kept at room temperature for 15 seconds and reweighed to obtain the mass after immersion in solutions (m2). The specimens were then replaced again in their tubes and stored in a desiccator with silica gel. Measurements during dehydration were performed again using the same methodology described in cycles of 24 hours to obtain the reconditioned constant mass, called ‘m3’.

The average sorption and solubility (mg/mm³) of each specimen was calculated according to the following equations: Sorption = m2 - m3 / V, Solubility = m1 - m3 / V, where m1 = mass after initial drying specimen (ug), m2 = mass after the immersion period in solutions (ug), m3 = final mass after drying (ug), V = volume in mm3. All measurements were performed with calibrated equipment by a single trained examiner and the final values were obtained plotting the formulas in an Excel sheet (Microsoft Office 2016).

-Statistical Analysis 

All data have a normal distribution of errors and were analysed by a two-way-ANOVA, considering the composite resins and mouthwashes as the main factors under study. A post-hoc Tukey test was used to compare means of sorption and solubility in studied factors. To evaluate the alcohol effect on sorption and solubility, data were grouped and paired by mouth rinses with alcohol and without alcohol and a paired t-test was performed. The SAS program version 9.0 was used to perform statistical tests with significance level set at 5%.

## Results

The 2-way-ANOVA showed significant effects for composite resins, mouthwashes and their interaction for both sorption and solubility results (*p* <0.05). The sorption results of the tested resins are shown in Table 3. For Filtek™ Bulk Fill resin, Listerine Cool Mint lead to a higher sorption than other rinses (*p* <0.05). For the X-tra Fil resin, it was observed that the Listerine Cool Mint and Periogard rinses, both containing alcohol in their composition, caused a higher degree of sorption than the other solutions (*p* <0.05), which did not differ from each other. Also, X-tra Fil showed statistically higher sorption values than Filtek™ Bulk Fill resin (*p* <0.05), except for Listerine Cool Mint, whose resins did not differ between each other (*p*> 0.05). For flow type bulk fill composites, Listerine Cool Mint (with alcohol) presented higher sorption in comparison to other mouthwashes (*p* <0.05). Furthermore, both low viscosity resins presented higher sorption than conventional viscosity resins, irrespective to the solutions used (*p* <0.05).

**Table 3:**
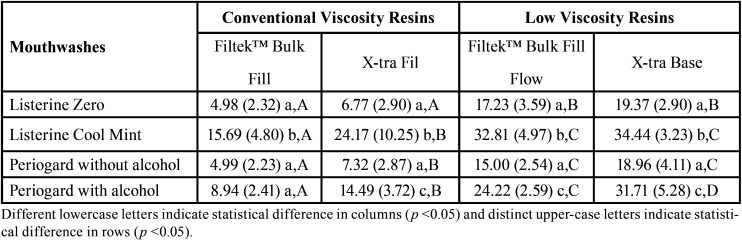
Mean (± SD) of sorption (μg/mm³) according to the resins and the mouthwases (n = 8).

Regarding solubility results (Table 4) of both kind of composites (conventional and low-viscosity), it was observed that Listerine Cool Mint (with alcohol) presented higher values when compared to the other mouthwashes, irrespective of the resin studied (*p* <0.05). In addition, the results showed that Filtek™ Bulk Fill presented statistically higher solubility values when compared to X-tra Fil (*p* <0.05). On the other hand, comparing low viscosity resins, X-Tra Base presented higher values of solubility when compared to the Filtek™ Bulk Fill Flow.

**Table 4:**
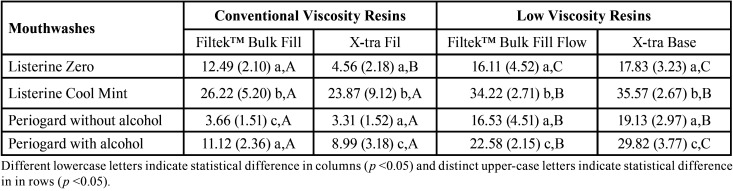
Mean (± SD) of solubility (μg/mm³) according to the resins and the mouthwases (n = 8).

**Figure 2:**
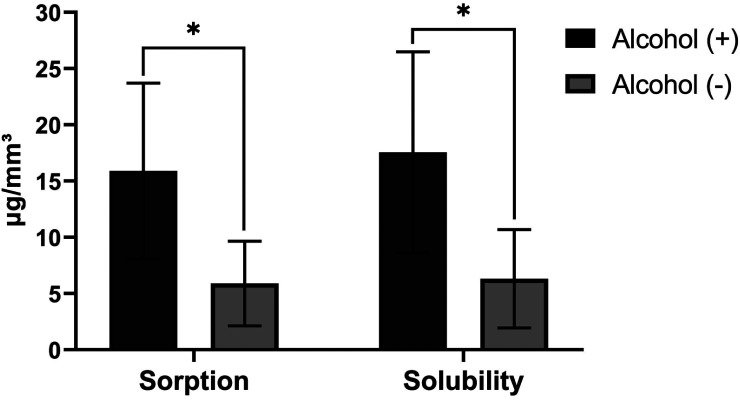
Sorption and Solubility (Mean ± SD, μg/mm³) of conventional viscosity bulk fill resins in the mouthwashes according to the presence (+) or absence (-) of alcohol (n = 32). The asterisk indicates a significant difference (*p* <0.05).

**Figure 3:**
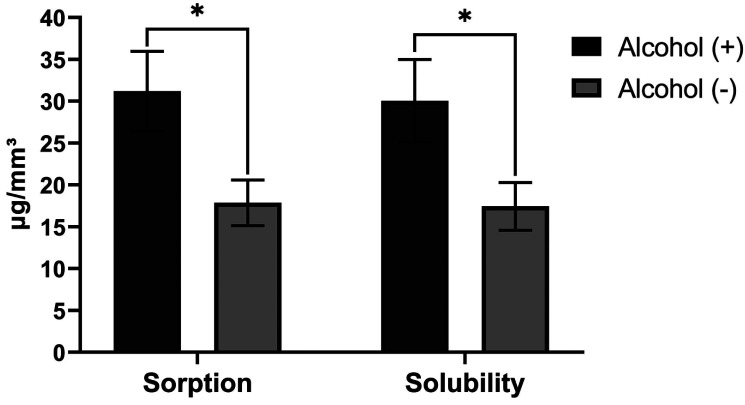
Sorption and Solubility (Mean ± SD, μg/mm³) of low viscosity bulk fill resins in the mouthwashes according to the presence (+) or absence (-) of alcohol (n = 32). The asterisk indicates a significant difference (*p* <0.05).

The alcohol-containing rinses in their composition led to higher values of sorption and solubility when compared to the non-alcohol solutions as can be observed in Figure 2 and 3 for conventional viscosity resins and low viscosity resins, respectively, where the data of each type of resins viscosity were grouped and paired with respect to the presence or absence of alcohol in the mouthwash.

## Discussion

The increasing amount of new dental materials on the market such as Bulk Fill resins brings the necessity to evaluate the behaviour of these materials in the oral environment challenges. These resins contain polymerisation modelers and may have the quality of the polymer network affected with consequently lower resistance to moisture when compared to the conventional composite resin (5). Thus, this study evaluated the effect of mouth rinses with and without alcohol on the degree of sorption and solubility of Bulk fill resins of different viscosities during a period of 7 days of storage.

The interaction of the resin composite with oral fluids occurs through the polymer chain separation by a molecule, which is not part of the primary polymer chemical bond (7). The water sorption causes external movement of the residual monomers and ions, causing solubility, which can trigger faults in the reaction of the components, especially in the monomers and also provoke silane hydrolysis, resulting in shrinkage, lower weight and reduction of the mechanical properties, reducing the durability of composite resins and formation of microgaps (8).

Several studies have been done on sorption and solubility of composite resins using different observation periods, solutions and specimens (20,21). However, there is a lack of studies with Bulk Fill resins immersed in mouthwashes, a product routinely prescribed by dentists for chemical biofilm control and that constantly interact with teeth and restorations.

The kinetics of the sorption process may be slower or faster according to the hydrophilic characteristic of resin matrix (5). Among the monomers evaluated in this study, the triethylene glycol dimethacrylate (TEGDMA) is the one with the highest hydrophilicity and higher sorption capacity (5), which could explain the worst performance of X-Tra Base compared to the other composite resins, since TEGDMA is in it chemical composition (Table 3). The other resins containing TEGDMA also have other monomers in their compositions such as UDMA or Bis-GMA, which are less hydrophilic than TEGDMA. Filtek™ Bulk Fill resin, whose composition contains Bis-EMA and UDMA, presented the lowest sorption of the evaluated materials. This can be explained by the fact that Bis-EMA present lower sorption and solubility in water, due to its hydrophobic and high conversion character (21).

Solubility is the measure of the amount of unconverted residual monomer that is released into the solution and may have the potential to impact the stability of the resin structure (3). The results of sorption and solubility of the same resin should be related, since the solvent needs to penetrate the polymer so that the leachable components can be released to the outside of the material (17). For conventional viscosity Bulk Fill resins, this relation was not observed, since both resins behave in a non-standardised way for the sorption and solubility tests (Table 4).

However, for low viscosity Bulk Fill resins, sorption and solubility results were similar (Table 4). For the more hydrophobic compounds, the lower sorption values were reflected at lower solubility values. Despite these results, other factors, such as the degree of conversion and the crosslinked network density may be more important in the sorption/solubility correlation (21).

It is known that hydrophilic materials present higher degradation by sorption and solubility than hydrophobic materials (20,21), however, hydrophobic matrices, such as Bis-GMA and UDMA, present in the composition of all resins evaluated, are also susceptible to chemical reactions by alcohol (21). Alcohol is considered a good solvent for the polymer chain of the resins and can cause a significant decrease in its properties and increase of composite wear in high concentrations. Alcohol is used in mouthwashes as a solvent, flavour enhancer and as an antiseptic agent (22).

The storage of the resin samples during 7 days in the different solutions showed that the alcohol-containing rinses led to a higher degree of sorption and solubility of the studied materials (Figures 2 and 3), especially Listerine Cool Mint, because it presents alcohol in greater concentration (approximately 30%). This can be explained because the ethanol penetrates the polymer network causing an expansion of the polymer structure, allowing the release of residual monomers and causing the dissolution of the linear polymer chain (23).

In Pereira *et al.* (18) study using similar methods, it was reported that Listerine Cool Mint also caused the highest degree of sorption for all composites tested compared to other rinses. In addition, it was reported that the mouth rinses Listerine and Periogard, both with alcohol in their composition, decreased the microhardness of composite resins (24).

According to ISO 4049 standard (19), for composite resins to be indicated as restorative materials, they must have a water sorption of less than 40 μg/mm³ and a solubility of less than 7.5 μg/mm³ for a period of 7 days of storage. The sorption values of all the resins were lower than the recommended values; while the solubility values of some resins were higher than those recommended, especially for solutions containing alcohol in their composition ( Table 3, Table 4).

## Conclusions

Despite the limitations of an *in vitro* study, it can be concluded that the sorption and solubility of the composites tested were higher in the alcohol-containing rinses in their composition. Thus, alcohol-free mouthwashes should be preferred in patients with extensive restorations, and there is a need for further *in vivo* studies. Low viscosity resins always presented worse results when compared to conventional viscosity resins. The composites that presented worse and better performance were X-Tra Base and Filtek™ Bulk Fill, respectively.
